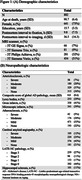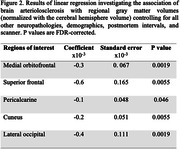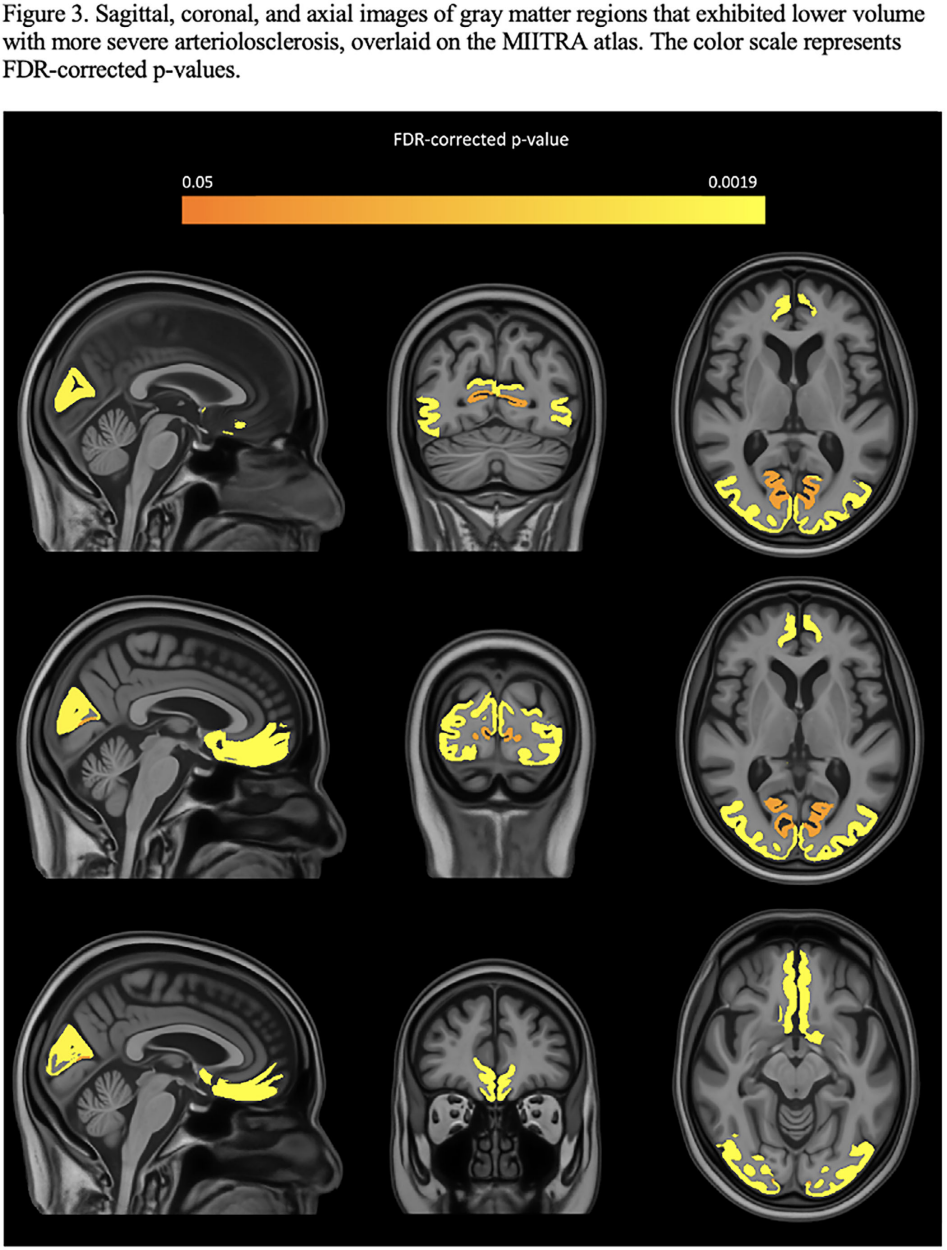# Brain arteriolosclerosis is linked to lower gray matter volume in community‐based older adults

**DOI:** 10.1002/alz.086662

**Published:** 2025-01-03

**Authors:** Ana Tomash, Mahir Tazwar, Md Tahmid Yasar, David A. Bennett, Julie A. Schneider, Konstantinos Arfanakis

**Affiliations:** ^1^ Illinois Institute of Technology, Chicago, IL USA; ^2^ Rush Alzheimer’s Disease Center, Rush University Medical Center, Chicago, IL USA; ^3^ Rush Alzheimer's Disease Center, Rush University Medical Center, Chicago, IL USA

## Abstract

**Background:**

Brain arteriolosclerosis is characterized by the thickening of vessel walls and arteriolar stenosis and is one of the primary pathologies of cerebral small vessel disease. Arteriolosclerosis is linked to lower cognitive and motor function, as well as an elevated risk of dementia. This study aimed to investigate the association of brain arteriolosclerosis with regional gray matter volumes in a large number of community‐based older adults.

**Method:**

Data from ex‐vivo MRI and detailed neuropathological evaluation were collected from older adults (N = 882) participating in four longitudinal, clinical‐pathologic cohort studies of aging (Rush Memory and Aging Project, Religious Orders Study, Minority Aging Research Study, and Clinical Core of the Rush Alzheimer’s Disease Research Center) (Fig. 1A). Cerebral hemispheres from all participants were obtained at autopsy and imaged ex‐vivo with a multi‐echo spin‐echo sequence (ME‐SE) on 3T clinical MRI scanners approximately one month postmortem. The acquired voxel size was 0.6mm × 0.6mm × 1.5mm, and the scan time was approximately 30 minutes. The gray and white matter were segmented in the ex‐vivo MRI data. Subsequently, the gray matter underwent further division into 42 cortical and subcortical regions using multi‐atlas segmentation. The volume of each region was measured and normalized by the participant’s cerebral hemisphere volume. Following ex‐vivo MRI, a detailed neuropathologic assessment was performed on pathologies including arteriolosclerosis, atherosclerosis, cerebral amyloid angiopathy, gross and microscopic infarcts, Alzheimer’s pathology, Lewy bodies, limbic‐predominant age‐related TDP‐43 encephalopathy neuropathological change (LATE‐NC), and hippocampal sclerosis (Fig. 1B). For statistical analysis, linear regression was employed to explore the association of brain arteriolosclerosis with regional gray matter volumes (normalized by cerebral hemisphere volume) controlling for all other neuropathologies (Fig. 1B), demographics (age at death, sex, years of education), postmortem intervals, and scanner (Fig. 1A).

**Result:**

More severe brain arteriolosclerosis was associated with lower volume in several gray matter regions, including medial orbitofrontal, superior frontal, pericalcarine, cuneus, and lateral occipital areas, independently of the effects of other neuropathologies (Figs. 2, 3).

**Conclusion:**

This study demonstrated that brain arteriolosclerosis is associated with lower volume in multiple gray matter regions independently of the effects of other vascular or neurodegenerative pathologies.